# Impact of orthodontic treatment on oral microbiome diversity and composition: A longitudinal study

**DOI:** 10.6026/973206300210062

**Published:** 2025-01-31

**Authors:** Mohammad Khursheed Alam, Mohammad Younis Hajeer, M. Alfaleh Muhanna Abdulrahim, Z. Alfaleh Ayman Falah, F. Alanazi Abdulmohsen Abdulkarim

**Affiliations:** 1 Department of Preventive Dentistry, College of Dentistry, Jouf University, Sakaka 72345, Saudi Arabia; 2 Department of Dental Research Cell, Saveetha Institute of Medical and Technical Sciences, Saveetha Dental College and Hospitals, Chennai 600077, India; 3 Department of Public Health, Daffodil International University, Dhaka 1207, Bangladesh; 4 Department of Orthodontics, University of Damascus, Damascus P.O. Box 16046, Syria

**Keywords:** Orthodontic treatment, oral microbiome, diversity, microbial composition, fixed appliances, longitudinal study

## Abstract

The change in oral microbiome diversity and composition during fixed orthodontic treatment was done using 16S rRNA sequencing. Saliva and plaque samples from 60 participants were analyzed at baseline, 3 months and 6 months. Alpha diversity significantly decreased at 3 months (mean: 2.8 ± 0.4) but partially recovered by 6 months (mean: 3.0 ± 0.3). Beta diversity analysis revealed significant microbial composition shifts (p < 0.01) with an increase in *Streptococcus mutans* and a decline in Streptococcus sanguinis. Hence, orthodontic treatment alters the oral microbiome, emphasizing the need for enhanced oral hygiene to prevent dysbiosis.

## Background:

The human oral cavity harbors a highly diverse and complex microbiome, consisting of over 700 species of bacteria, fungi, viruses and archaea, which play a pivotal role in maintaining oral and systemic health [1]. Dysregulation has been linked to a wide range of oral diseases, including dental caries, periodontal disease and systemic conditions such as cardiovascular diseases and diabetes [2]. Orthodontic treatment, particularly with fixed appliances, is a well-established therapeutic modality for correcting malocclusion and improving dental aesthetics and function. However, the introduction of fixed orthodontic appliances significantly alters the oral environment, creating new niches that favor microbial colonization and biofilm formation [3]. Fixed orthodontic appliances increase the complexity of oral hygiene due to the presence of brackets, wires and bands, which provide retention sites for biofilm accumulation and disrupt the natural clearance of microorganisms by saliva and oral hygiene practices [4]. This alteration may predispose individuals to an increased risk of enamel demineralization, gingivitis and periodontal disease during the course of treatment [5]. The resulting changes in the oral microbiome are characterized by a shift from a symbiotic to a dysbiotic state, marked by an increase in acidogenic and cariogenic bacterial species such as *Streptococcus mutans* and Lactobacillus spp [6]. Emerging studies utilizing high-throughput sequencing methods, such as 16S rRNA gene sequencing, have provided valuable insights into the composition and diversity of the oral microbiome during orthodontic treatment. These techniques allow for the identification of both cultivable and non-cultivable microorganisms, enabling a comprehensive understanding of microbial dynamics over time [7]. However, there remains a paucity of longitudinal studies exploring the temporal effects of orthodontic treatment on oral microbiome diversity and composition, especially in diverse populations. Understanding these changes is critical for developing targeted interventions to mitigate adverse effects associated with microbial dysbiosis during orthodontic therapy. Therefore, it is of interest to investigate the longitudinal impact of fixed orthodontic treatment on the diversity and composition of the oral microbiome using advanced sequencing techniques, contributing to the existing body of knowledge in this area and informing better clinical practices.

## Materials and Methods:

This longitudinal study included 60 participants, aged 12-18 years, undergoing fixed orthodontic treatment at a tertiary dental care center. Ethical clearance was obtained from the institutional ethics committee and informed consent was secured from all participants and their guardians. Individuals with systemic conditions, ongoing antibiotic therapy, or a history of periodontal disease were excluded from the study. Saliva and plaque samples were collected at three time points: baseline (prior to appliance placement), 3 months and 6 months after treatment initiation. Samples were collected under standardized conditions using sterile swabs for plaque and expectoration into sterile containers for saliva. All samples were immediately stored at - 80°C until DNA extraction. Microbial DNA was extracted using a commercial DNA extraction kit following the manufacturer's protocol. The V3-V4 region of the 16S rRNA gene was amplified by polymerase chain reaction (PCR) using universal bacterial primers and sequencing was performed on a high-throughput Illumina MiSeq platform. The sequencing data were processed using QIIME2 software for quality filtering, chimera removal and taxonomic assignment against the SILVA database. Alpha diversity was assessed using the Shannon and Simpson indices, while beta diversity was analyzed using principal coordinate analysis (PCoA) and permutational multivariate analysis of variance (PERMANOVA). Statistical analysis was conducted using SPSS software, with significance set at p < 0.05. This methodology enabled the evaluation of temporal changes in oral microbiome diversity and composition associated with orthodontic treatment.

## Results:

The study evaluated the impact of fixed orthodontic treatment on oral microbiome diversity and composition over a 6-month period. Changes in alpha and beta diversity, as well as shifts in microbial composition, were observed and are summarized below.

## Alpha diversity:

The Shannon Index showed a significant decrease in microbial diversity at 3 months (mean: 2.8 ± 0.4) compared to baseline (mean: 3.2 ± 0.3), followed by partial recovery at 6 months (mean: 3.0 ± 0.3). Similarly, the Simpson Index indicated reduced diversity at 3 months (mean: 0.84 ± 0.05) relative to baseline (mean: 0.88 ± 0.04). The trends for alpha diversity metrics are shown in Table 1.

## Beta diversity:

Principal coordinate analysis (PCoA) based on Bray-Curtis dissimilarity revealed distinct clustering at 3 months, indicating a significant shift in microbial composition compared to baseline (PERMANOVA, p < 0.01). By 6 months, the clustering pattern suggested a partial return towards the baseline state but remained distinct.

## Microbial composition:

Relative abundance analysis showed a notable increase in *Streptococcus mutans* from 18% at baseline to 30% at 3 months, with a slight decrease to 25% at 6 months. In contrast, *Streptococcus sanguinis*, a commensal species, decreased from 22% at baseline to 15% at 3 months and partially recovered to 18% at 6 months. The relative abundance of *Porphyromonas gingivalis* and *Prevotella intermedia*, associated with periodontal disease, increased during the treatment period. Details of microbial shifts are presented in Table 2. These results underscore significant but reversible changes in oral microbiome diversity and composition during fixed orthodontic treatment.

## Discussion:

This study highlights the significant impact of fixed orthodontic treatment on the oral microbiome, with noticeable alterations in microbial diversity and composition over a 6-month period. The findings align with previous studies that have demonstrated orthodontic appliances as contributors to microbial dysbiosis by creating niches favorable for biofilm accumulation [1, 2]. These changes have clinical implications, particularly for the risk of enamel demineralization, gingivitis and other oral health issues during orthodontic therapy. The observed reduction in alpha diversity during the initial 3 months of treatment reflects a disruption in the microbial balance, consistent with findings in earlier research [3, 4]. Fixed appliances hinder oral hygiene practices, allowing the proliferation of acidogenic and cariogenic species. Partial recovery of diversity at 6 months suggests microbial adaptation to the new oral environment, which is a key area for further exploration [5]. The beta diversity analysis revealed significant shifts in microbial composition at 3 months, corroborating studies that reported distinct microbial clustering shortly after appliance placement [6, 7]. The increased relative abundance of *Streptococcus mutans* and Lactobacillus spp. during the early stages of treatment is well-documented, as these species thrive in acidic environments and contribute to enamel demineralization [8, 9]. The partial recovery of *Streptococcus sanguinis* by the 6-month mark is encouraging, as it indicates the potential for re-establishing microbial equilibrium with proper oral hygiene interventions [10]. Furthermore, the rise in periodontal pathogens such as *Porphyromonas gingivalis* and *Prevotella intermedia* highlights the importance of monitoring periodontal health during orthodontic treatment [11, 12]. The findings emphasize the critical role of stringent oral hygiene practices and regular professional care during orthodontic treatment to mitigate the risk of microbial dysbiosis. Adjunctive strategies such as antimicrobial mouthwashes, probiotic supplementation and improved dietary practices could play a significant role in managing the microbial shifts [13, 14]. Personalized oral hygiene protocols tailored to the individual's microbiome profile may further enhance outcomes during orthodontic therapy. This study was limited by its sample size and single-center design, which may affect the generalizability of the findings. Additionally, the study did not account for the potential influence of dietary and behavioral factors on microbial changes. Future research should focus on larger, multicenter studies to validate these findings and explore the long-term effects of orthodontic treatment on the oral microbiome. Investigating the role of advanced interventions, such as microbiome-modulating agents and artificial intelligence-based predictive models, could provide new avenues for improving patient outcomes [15].

## Conclusion:

Fixed orthodontic treatment significantly alters oral microbiome diversity and composition, particularly in the early stages of therapy. This data underscore the need for proactive measures to mitigate microbial dysbiosis and maintain oral health during orthodontic treatment.

## Figures and Tables

**Table 1:** Changes in alpha diversity metrics over time

**Time Point**	**Shannon Index (Mean ± SD)**	**Simpson Index (Mean ± SD)**
Baseline	3.2 ± 0.3	0.88 ± 0.04
3 Months	2.8 ± 0.4	0.84 ± 0.05
6 Months	3.0 ± 0.3	0.86 ± 0.05

**Table 2:** Relative abundance of key microbial species (%)

**Species**	**Baseline (%)**	**3 Months (%)**	**6 Months (%)**
*Streptococcus mutans*	18	30	25
*Streptococcus sanguinis*	22	15	18
*Porphyromonas gingivalis*	5	8	7
*Prevotella intermedia*	4	7	6
